# Abdominal aortic peripheral intervention to facilitate intra-aortic balloon pump support during high risk percutaneous coronary intervention: a case report

**DOI:** 10.1186/s12872-015-0013-5

**Published:** 2015-03-10

**Authors:** See W Low, Justin Z Lee, Kwan S Lee

**Affiliations:** Department of Cardiovascular Diseases, University of Arizona South Campus, 2800 E Ajo Way, Tucson, AZ 85713 USA; Department of Internal Medicine, University of Arizona, 1501 N Campbell Ave, RM 6336, Tucson, AZ 85724 USA; 3950 S Country Club Road, Suite 200, Tucson, AZ 85714 USA

**Keywords:** Abdominal aortic stenosis, Intra aortic balloon pump, Percutaneous coronary intervention

## Abstract

**Background:**

The use of intra-aortic balloon pump (IABP) via the trans-femoral approach has been established for hemodynamic support in patients undergoing high-risk percutaneous coronary intervention (PCI). However, there are various challenges associated with its use, especially in patients with aortoiliac occlusive arterial disease.

**Case presentation:**

We describe a case of high-risk PCI with IABP support complicated by intra-procedural detection of severe abdominal aortic stenosis that was successfully overcome with angioplasty of the stenotic lesion.

**Conclusions:**

Our report highlights distal abdominal aortic stenosis as a potential barrier to successful PCI with IABP support, and angioplasty as an effective means to overcome it.

## Background

The use of intra-aortic balloon pump (IABP) is a well–recognized technique for hemodynamic support in patients undergoing high-risk percutaneous coronary intervention (PCI) [1]. The trans-femoral approach has been the preferred percutaneous access for IABP insertion. However, this approach has its own challenges, particularly in patients with severe occlusive arterial disease [2]. We describe a case of high-risk PCI with IABP support complicated by intra-procedural detection of severe abdominal aortic stenosis, and the approach we used to overcome it.

## Case presentation

A 68-year-old man with history of coronary artery disease, ischemic cardiomyopathy, diabetes mellitus, primary prevention automated implantable cardioverter defibrillator (AICD), polio, obesity and systolic heart failure presented to our institution with increasing ischemic chest pain for three days. He was found to have a non-ST segment elevation myocardial infarction with decompensated heart failure with ejection fraction of 24%, as well as acute kidney injury secondary to cardiorenal syndrome. This was also complicated by sustained slow ventricular tachycardia (VT), which caused further decompensation.

**Figure 1:**
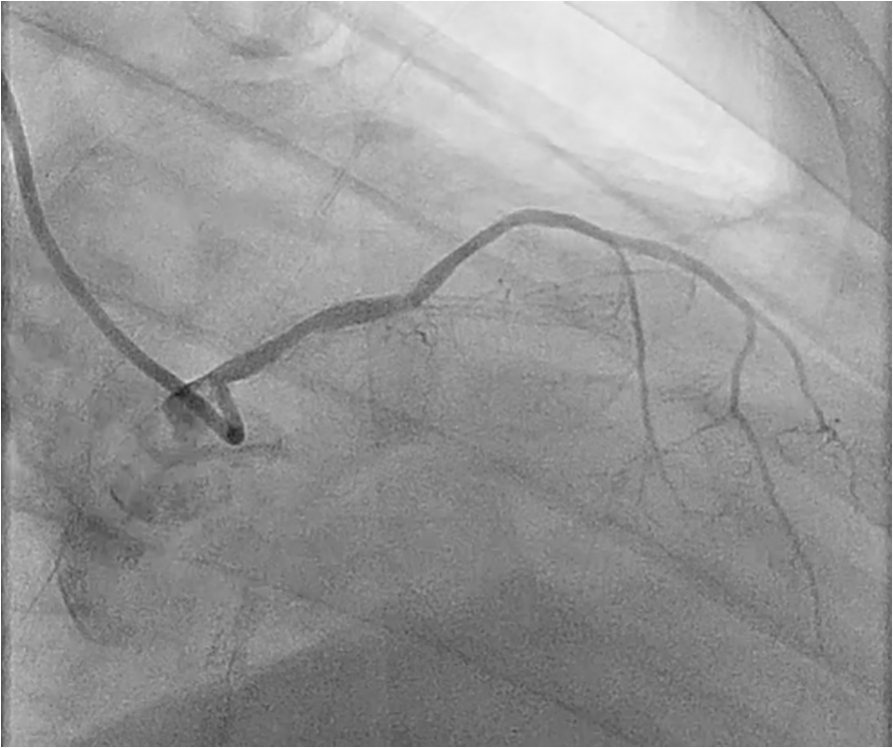
**Coronary angiography before PCI showing 70%** **in**-**stent restenosis** **(arrow)** **of the distal left main coronary artery extending into the LAD with chronic occlusion of ostial circumflex and mid LAD just after origin of small first diagonal.**

**Figure 2:**
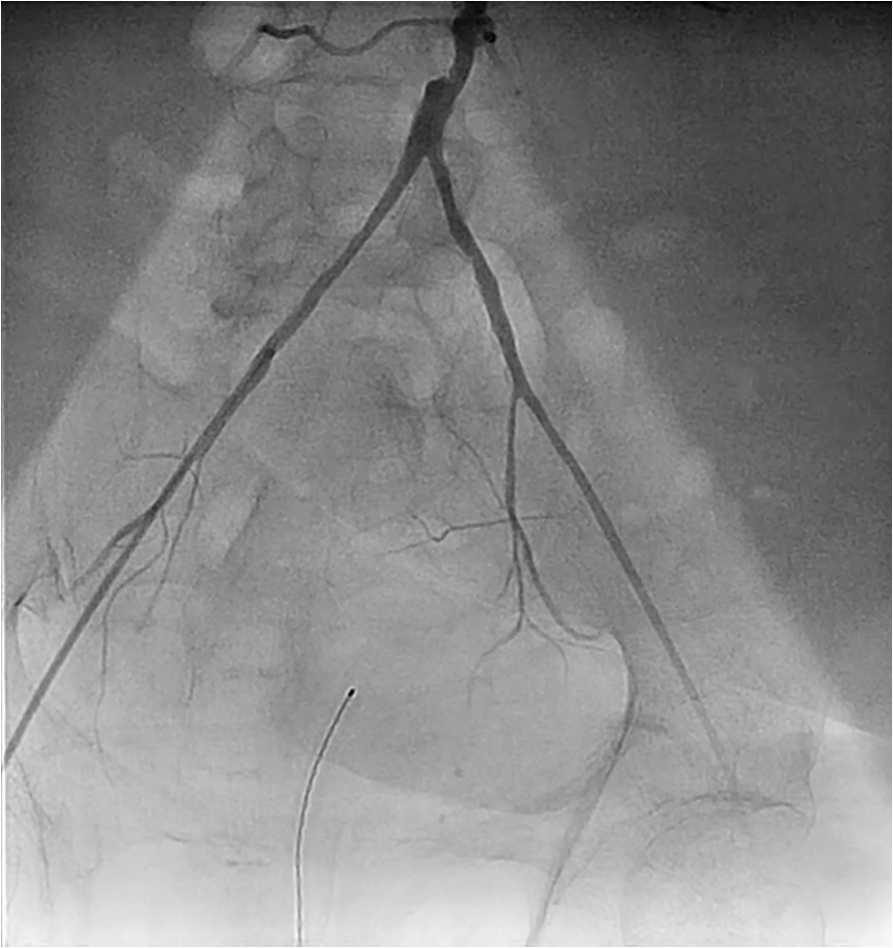
**Distal abdominal aortic angiography with bilateral iliofemoral runoff showing distal abdominal aortic, focal 80% eccentric stenosis with patent, small caliber bilateral ilio-femoral arterial system, excluding possible use of Impella percutaneous left ventricular assist device via femoral approach.**

**Figure 3:**
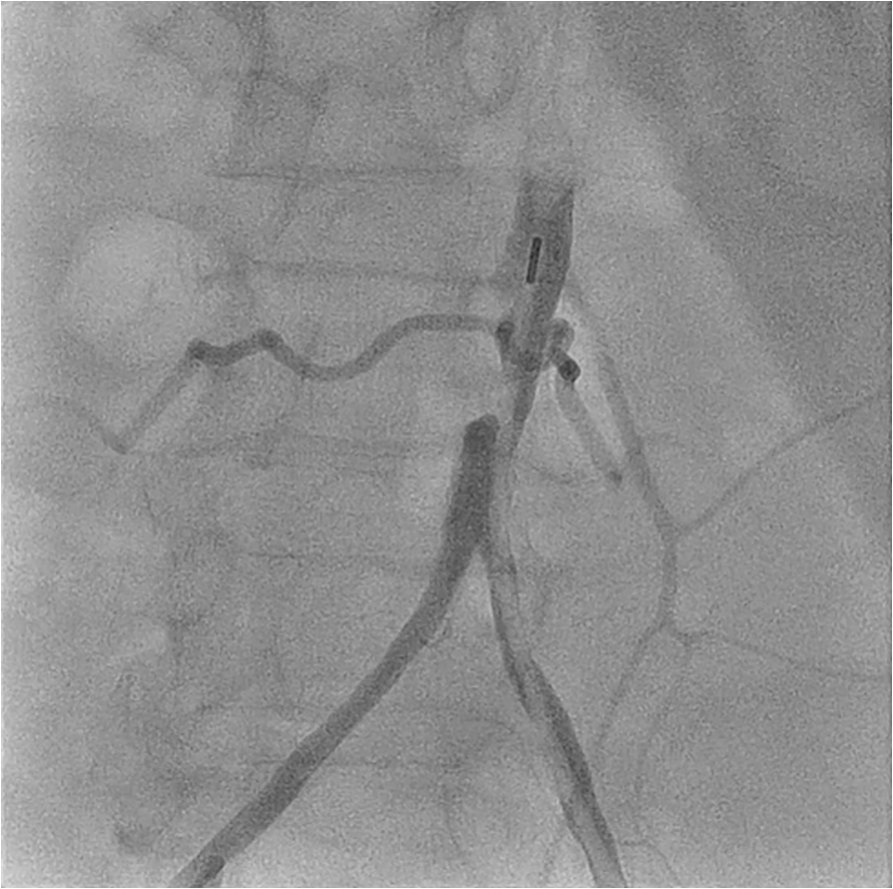
**Post insertion of IABP in the abdominal aorta, showing limited space for possible adjacent guide catheter passage.**

**Figure 4:**
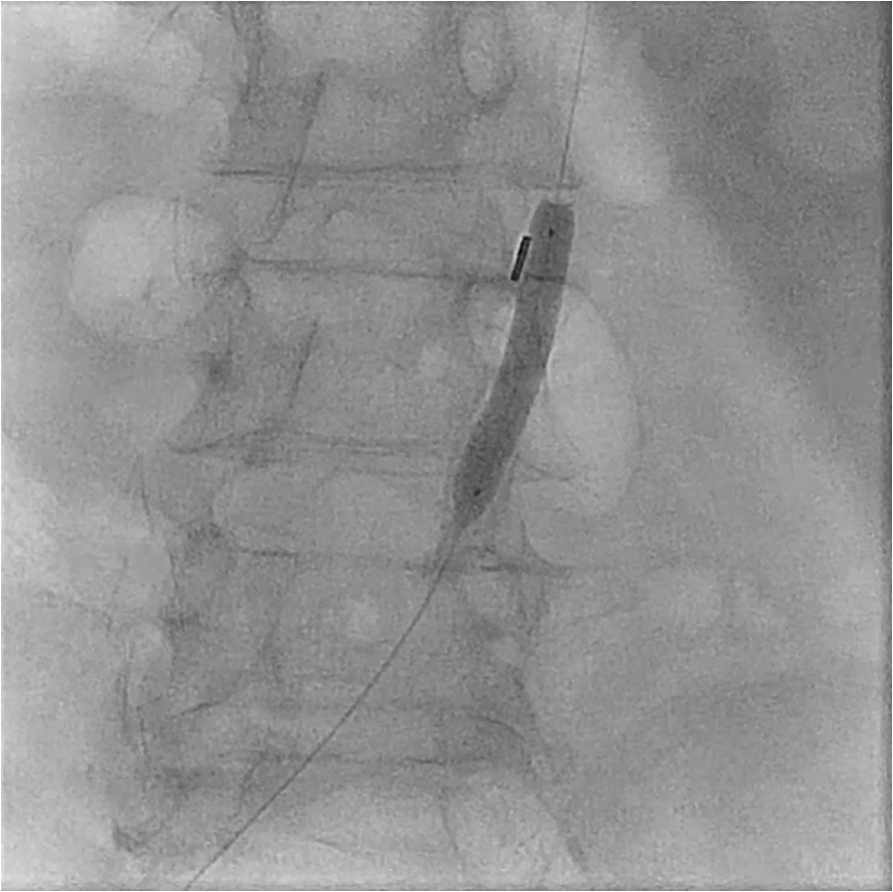
**Angioplasty of abdominal aortic stenosis with IABP in**-**situ utilizing Viatrec 7.0 × 40 mm balloon.**

**Figure 5:**
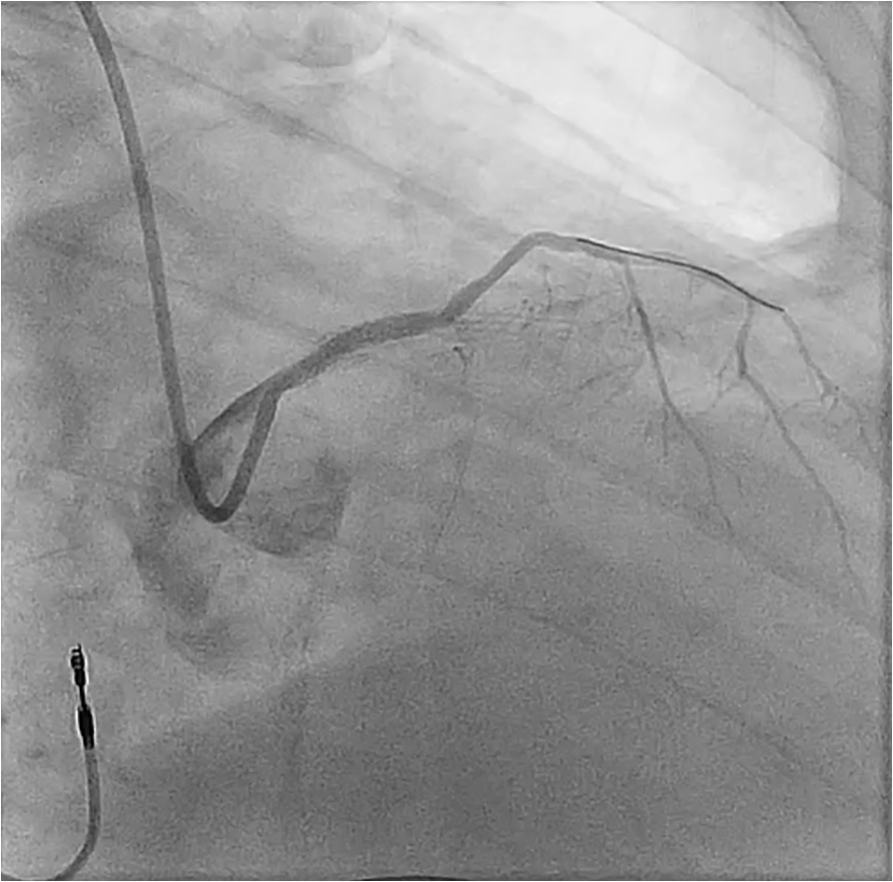
**Post PCI of the left main coronary artery with placement of Xience Xpedition 2.5 × 15 mm DES.**

A decision was made to perform emergency cardiac catheterization with possible PCI. No good radial pulses were palpable. Secondary to his underdeveloped lower extremities from polio, obesity and faint femoral pulses, micropuncture technique under fluoroscopic guidance was used to obtain retrograde right common femoral arterial access, with placement of a 6-French sheath. Selective left coronary angiography was performed with a JL4 catheter, revealing a distal left main into ostial left anterior descending artery (LAD) 70% in-stent restenosis (ISR) lesion which gave rise to a small diagonal after which the LAD was chronically occluded, and a chronically occluded circumflex (Figure 1). This resulted in significant hypotension from severe ischemia and the development of cardiogenic shock. Distal abdominal aortography was performed using a 6-French JR4 catheter with bilateral iliofemoral runoff to determine if he was a candidate for Impella (Abiomed, Danvers, Massachusetts) percutaneous left ventricular assist device or IABP support and to outline the course of his femoral artery for contralateral groin access (Figure 2). Utilizing micropuncture technique, left common femoral arterial access was gained with placement of an 8-French sheath. A 40cc Datascope IABP was advanced via the left common femoral access into the appropriate position, with difficulty during initial wiring with the 0.028” wire secondary to a distal abdominal aortic lesion (Figure 3). IABP support was commenced at 1:1 ratio with vasopressor and inotropic support. From the right common femoral access site, a 0.035” J-wire or angled Glide wire (Terumo Interventional Systems, Somerset, New Jersey) could not get pass the abdominal aortic lesion with the IABP in situ. Thus, the distal abdominal aortic lesion was crossed with a 0.014” Runthrough coronary guidewire (Terumo Interventional Systems, Somerset, New Jersey) and the lesion was dilated with a Viatrec (Abbott, Abbott Park, Illinois) 7.0 × 40 mm balloon to 7 atm for 30 seconds serially (Figure 4). A 5-French Glidecath was then used to exchange the Runthrough for an exchange length 0.035” J-wire in the ascending aorta. A 6-French JR4 was used to perform the selective right coronary angiography. Intravenous heparin was used as the procedural anticoagulant. The left main was engaged with a 6-French EBU 3.5 side hole guide, providing good support. The diagonal was wired with 0.014” Runthrough guidewire and the left main into ostial LAD ISR was directly stented with a Xience Xpedition 2.5 × 15 mm drug eluting stent (DES) (Abbott, Abbott Park, Illinois) to 20 atm for 10 seconds (Figure 5). Subsequently a NC Trek (Abbott, Abbott Park, Illinois) 3.0 × 12 mm balloon was used to post-dilate to 24 atm for 10 seconds.

**Figure 6:**
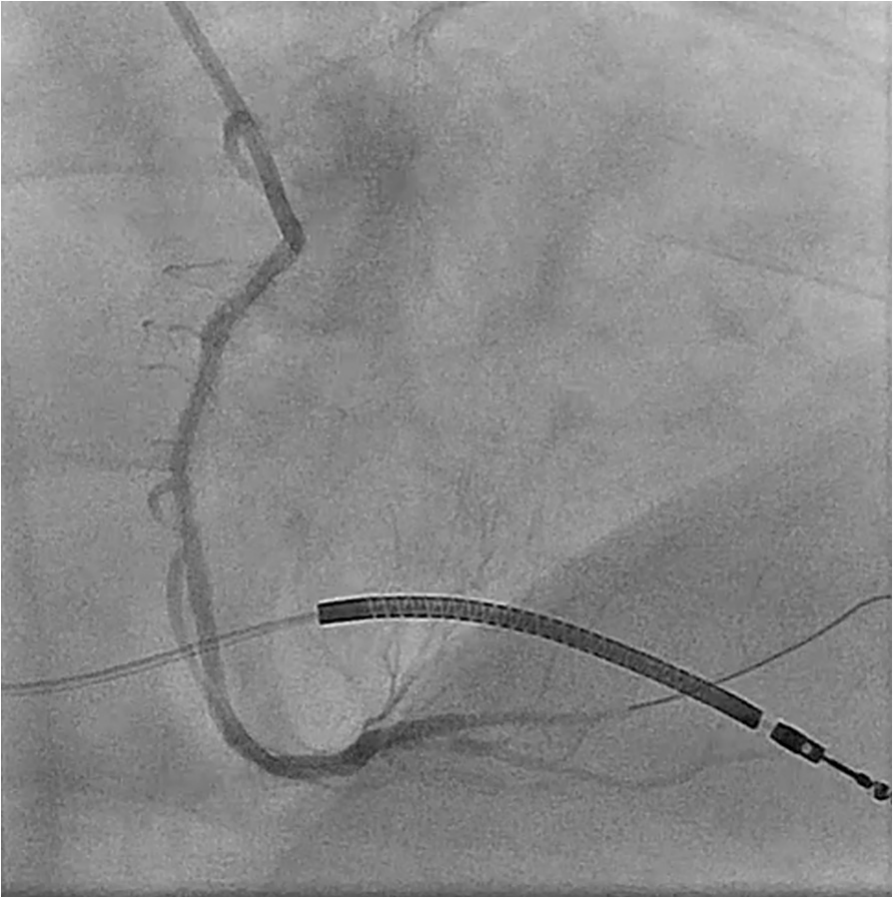
**Post PCI of the RCA**, **distally with placement of overlapping Xience Xpedition 2.25 × 18 mm and 2.5 × 12 mm DES and proximally with overlapping Xience Xpedition 3.0 × 38 mm and 3.0 × 15 mm DES.**

Post PCI, he developed recurrent slow VT and this was cardioverted with anti-tachycardia pacing via his implantable cardiac defibrillator (ICD). His right coronary artery (RCA) was then engaged with a 6-French AR1 guide with side holes and the posterolateral branch (PLB) was wired with the Runthrough guidewire. Overlapping Xience Xpedition 2.25 × 18 mm and 2.5 × 12 mm DES were deployed into the distal right, followed by proximally overlapping Xience Xpedition 3.0 × 38 mm and 3.0 × 15 mm DES for his mid to ostial ISR (Figure 6). Final coronary angiographic images were obtained.

Distal abdominal aortography digital subtraction angiography (DSA) was then performed with the AR1 guide and repeat dilatation was then performed with an Armada 9.0 × 80 mm balloon (Abbott, Abbott Park, Illinois) to 12 atm for 30 seconds. Secondary to concerns about limb ischemia, the IABP and 8-French sheath were both removed and manual pressure was held for 30 minutes for hemostasis. He made a good initial clinical recovery without any acute post-procedural complications such as bleeding requiring blood transfusion or pseudo-aneurysm. However, he had continued deterioration of his renal function due to acute tubular injury from his previous episodes of hypotension and cardiorenal syndrome, to the point where dialysis was required. The patient and family decided against dialysis after several days, and the patient subsequently succumbed to his renal failure.

## Discussion

We describe a case of angioplasty of severe abdominal aortic stenosis to facilitate successful emergency high-risk PCI with IABP support complicated by intra-procedural detection of the abdominal aortic stenosis. To our knowledge, no similar case report has been published. The benefit of IABP for hemodynamic support has been established in patients undergoing high risk PCI [1], complicated by cardiogenic shock [3]. The common preferred site for percutaneous insertion of IABP is the common femoral artery, and the distal end of the catheter is advanced and positioned in the proximal descending aorta. Consequentially, peripheral aortoiliac vascular disease has been described as a relative contraindication to IABP insertion, although several approaches have been described to overcome utilizing peripheral interventional techniques especially with iliac disease [4-6].

Abdominal aortic stenosis has an overall incidence of 1 in 62,500 [7]. The treatment of abdominal aortic stenosis ranges from surgical therapy, with aortoiliac endarterectomy or aortobifemoral bypass, to endovascular therapy, with balloon angioplasty or stenting [8]. In our case, we discovered the abdominal aortic stenosis during emergency high-risk PCI after having difficulty with passing the 0.032” J-wire through a section of the distal abdominal aorta with the IABP in-situ. We proceeded with angioplasty of the stenosis, which allowed for passing of the J-wire over the lesion with the IABP in-situ.

Other methods of IABP insertion include trans-radial, trans-axillary, trans-brachial method, and trans-aortic method. Burack at al’s and H’Doubler et al’s have reported the use of transaxillary or transaortic approach. However, in patients who are hemodynamically unstable, this approach is not appropriate, as it requires general anesthesia and surgical procedures [9,10]. We selected the trans-femoral approach over the trans-radial approach because of the critical emergent need for IABP support and the lack of palpable radial pulses in our patient. There is limited data on the superiority of trans-radial approach in critical situation. In the RADIAL PUMP UP trial, it is shown that the use of transradial approach in high risk patients who is undergoing PCI and requires IABP support is associated with fewer thirty days net adverse clinical events (NACEs) of postprocedural bleeding, cardiac death, myocardial infarction, target lesion revascularization and stroke, mainly because of lower access-related bleeding events although there is no significant differences in the hospital stay length [11]. In a retrospective study, the use of transbrachial versus transfemoral was compared and it only showed the use of transbrachial to have reduced bedrest time (0 minutes vs 340.0 ± 104.9 minutes; *P* < .001) and duration of hospital stay (1.4 ± 0.6 days vs 5.4 ± 7.1 days; *P* = .04) [12]. The use of transbrachial approach has not been widely reported as the small diameter of the brachial artery requires smaller devices and has the potential to increase the vascular compromise to the distal vasculature.

The most common complications of IABP use are vascular complications such as ischemia, vascular obstruction and hemorrhage [13,14]. Established risk factors for bleeding in patients with IABP includes female gender, peripheral vascular disease, diabetes, body surface area (<1.65 m^2^), age (>75 years), and aggressive anticoagulant regimens (eg, glycoprotein IIb/IIIa inhibitor) [15]. Our patient is a 68-year-old man with multiple risk factors namely, history of coronary artery disease, diabetes mellitus, obesity and systolic heart failure. However, he did not suffer from any post-procedural bleeding complications.

The data on the use of IABP in patients post myocardial infarction with cardiogenic shock remains inconclusive. IABP SHOCK-II trial was the first large-scale multicenter randomized trial of balloon pump–supported early revascularization in AMI complicated by cardiogenic shock. No significant differences in the 30-day mortality and secondary outcomes were found. Although the timing, clinical scenarios and conditions which IABP demonstrates its beneficial effects remain unclear and despite the randomized controlled trial data suggesting no IABP-supported revascularization in post-myocardial infarction cardiogenic shock patients, the IABP insertion should still be tailored to the clinical condition of the patient and the caution of the operating physician [16].

## Conclusions

This report highlights severe distal abdominal aortic stenosis as potential challenge faced when attempting PCI with IABP support via the trans-femoral approach, and the utilization of angioplasty of the stenosis as a means to overcome it.

## Consent

Written informed consent was obtained from the patient’s next of kin for publication of this case report and any accompanying images. A copy of the written consent is available for review by the Editor of this journal.

## Abbreviations

IABP: Intra-aortic balloon pump
PCI: Percutaneous coronary intervention
AICD: Automated implantable cardioverter defibrillator
VT: Ventricular tachycardia
LAD: Left anterior descending artery
ISR: In-stent restenosis
DES: Drug eluting stent
ICD: Implantable cardiac defibrillator
RCA: Right coronary artery
DSA: Digital subtraction angiography
